# Psychosocial Interventions for Patients with Severe COPD—An Up-to-Date Literature Review

**DOI:** 10.3390/medicina55090597

**Published:** 2019-09-16

**Authors:** Marta Rzadkiewicz, Jacek Nasiłowski

**Affiliations:** 1 Department of Medical Psychology and Medical Communication, Medical University of Warsaw, 00-575 Warsaw, Poland; 2 Department of Internal Medicine, Pulmonary Diseases and Allergy, Medical University of Warsaw, 02-097 Warsaw, Poland

**Keywords:** long-term oxygen therapy, non-invasive ventilation, respiratory failure, psychosocial intervention, well-being, mental health, HRQoL, COPD

## Abstract

*Background and Objectives*: Chronic obstructive pulmonary disease (COPD) is a life limiting condition with a long list of serious psychosocial consequences, aggravating with illness progression. In advanced stages, chronic respiratory failure often develops, which might undermine mental health and reduce activity. The study objective was to review the recent studies concerning psychosocial interventions dedicated to patients with severe COPD. *Materials and Methods*: The PubMed database was searched for terms, such as ‘COPD and long-term oxygen therapy, non-invasive ventilation, severe or respiratory failure’ and ‘psychological or psychosocial or mental health and intervention.’ Studies were included that described patients with stable, severe COPD and the outcomes of psychosocial interventions. Results and *Conclusions*: Thirty-four studies were identified and divided into four thematic groups: home medical support, exercise, self-management and mental health. The number of studies that focused on mental health preservation in severe COPD was very limited; i.e., none refer directly to those treated with respiratory failure. Improving patients’ self-efficacy gave promising effects to the acceptance of palliative care, pulmonary rehabilitation completion and mental health. Physical activity might be recommended to be included in interventions for mental health enhancement, although little is known about the role of the particular forms of exercise. An increasing beneficial use of new technologies for psychosocial interventions was noted. Psychosocial interventions applied in advanced COPD underline the roles of self-efficacy, telehealth and physical activity in physical and mental health preservation. However, all of the above elements need to be independently tested on more homogenous groups of patients and have the possible modes of their treatment analysed.

## 1. Introduction

Chronic airflow limitation in chronic obstructive pulmonary disease (COPD) usually comes along with comorbidities and extrapulmonary manifestations which are significant predictors of disease burden and survival [1]. Innovative therapies and care give the patients a chance to live longer with COPD, hence the group of patients with advanced COPD is growing in number and in age. The variety of phenotypes of COPD requires an individual approach and an interdisciplinary management; going beyond the lungs, and including the careful monitoring of the patient’s psychological functioning is necessary [2,3]. As time passes, pulmonary symptoms and/or comorbidities often accumulate, due to both COPD progression and the patient’s ageing, making the treatment even more challenging in advanced stages. Typically, advanced COPD stages are marked with increasing exertional dyspnoea; a lack of energy, resulting in serious limitations to everyday activities; deprivation of basic needs; and a deteriorating quality of life. In the Global Initiative for Chronic Obstructive Lung Disease (GOLD) progression classification, the meaning of the above symptoms was recently emphasized [4]. In the course of the degradation of the lung tissue and airflow limitations, the gas exchange impairment increases and chronic respiratory failure develops. The introduction of long-term oxygen treatment (LTOT) and home non-invasive ventilation (NIV) may improve survival and have the potential to influence health related quality of life (HRQoL) [5,6] Nevertheless, too often patients feel restrained by and dependent on LTOT/NIV [7]. Not surprisingly, emotional problems [8] or worsening of spiritual well-being [9] is coincident with illness progression. Hence, there is an urgent need for evidence-based psychosocial interventions in this population.

Aim: This study sought to explore the recently published findings investigating psychosocial intervention programs concerning severe COPD, particularly with chronic respiratory failure.

Psychosocial intervention in this review is understood in accordance with a definition of psychosocial care. It is concerned with “the psychological and emotional well-being of the patient and their family and carers, including issues of self-esteem; insight into, and adaptation to the illness and its consequences; communication; social functioning; and relationships” [10] (Dix and Glickman, 1997). Of note, this formulation requires psychosocial intervention to be perceived more in terms of the outcomes than the methods used. Caring activities centred on the alleviation of symptoms, and those that aim to cope with comorbidities or to increase self-management can be thus considered. Meanwhile, appropriate care regarding psychosocial needs and quality of life maintenance, especially in advanced COPD with oxygen/ventilatory aid, appears unrecognized [11,12]. In a review investigating qualitative studies on the needs of patients with COPD, which encompassed more than 20 years of research and five databases [11], the authors identified only eight studies, which included 108 patients, of which only 15 participants were on oxygen treatment.

### Case Vignette

A 70-year-old man, a former smoker (40 pack-years) with severe COPD (forced expiratory volume in the first second (FEV1) 0.54 mL, 18% predicted value). Comorbidities: arterial hypertension and cachexia (BMI 19 kg/m^2^). Last 36 months on LTOT; currently using oxygen 24 h per day. Gradual development of severe hypercapnia with concomitant symptoms was noted and the decision about long-term NIV was set up in February 2018. During hospitalization, symptoms of anxiety and depression occurred, which aggravated respiratory symptoms. Quetiapine, 75 mg/day, was introduced, with a moderate improvement.

On admission to a home mechanical ventilation centre, the patient reported mild breathlessness at rest and severe exercise intolerance, MRC 5. Long-term NIV was administered by a face mask in a spontaneous/timed mode. The inspiratory pressure was progressively increased to reach the value of 22 cmH_2_O, 10–12 h/day, after 3 months of treatment. Arterial blood gas analysis on oxygen, 1.5 L/min via nasal cannula, showed satisfying results: pH 7.45, PaCO_2_ 46 mmHg and PaO_2_ 66 mmHg. In spite of improvements in regard to respiratory failure, the patient complained of frequent attacks of sudden breathlessness aggravated by a sensation of fear and panic. He was afraid of performing any physical activity and spent most of the time in a chair with fixed upper limbs.

The patient benefits to some extent from psychiatric pharmacotherapy, and still recently requests to double the doses of his anxiolytics, as the frequency of his irritability and anxiety symptoms has increased. He fosters his passion for street art, which helps keep him fighting with the illness, apart from his much-appreciated family support. Despite being cared for by a team of qualified specialists, this patient’s main concern is not addressed. He might significantly benefit from a specialist psychosocial intervention in terms of mood, perceived control, self-management, alleviation of psychosocial burden and health related quality of life improvement. The above ailments are not uncommon, yet there are no standard recommendations; therefore, a review was performed in the aim to search for potentially effective, psychosocial solutions.

## 2. Materials and Methods

A literature search was undertaken in order to identify recent investigations that assessed interventions focused on psychosocial support for patients with advanced COPD, including those with chronic respiratory failure.

### Inclusion and Exclusion Criteria

A two-step search of the PubMed database for peer-reviewed articles published in English between 2009 and February 2019 was performed. The first step was focused on the following terms:

’COPD’ in the title/abstract and ‘long-term non-invasive ventilation’ or ‘home mechanical ventilation’ or ‘long-term oxygen treatment’ anywhere in the text;

Intervention and ‘psychological’ or ‘psychoeducation’ or ‘psychosocial’ or ‘quality of life’ or ‘depression’ or ‘anxiety’ or ‘distress’ or ‘well-being’ or ‘cognitive behavioural therapy’ in the title/abstract.

Since the output showed 48 records, but none indicated relevant research with homogenous samples of patients treated with LTOT/NIV, the second step with a different set of keywords (based on the available literature search) was performed for:

COPD and ‘advanced’ or ‘severe’ or ‘end of life’ or ‘end stage’ or ‘respiratory failure’ in the title/abstract; and ‘intervention’ and ‘psychological’ or ‘psychoeducation’ or ‘psychosocial’ or ‘quality of life’ or ‘depression’ or ‘anxiety’ or ‘distress’ or ‘well-being’ or ‘cognitive behavioural therapy’.

The keywords were selected to enable a choice of interventions that could be considered for recommendation. This search resulted in 208 records, with 66 eligible titles, of which 24 articles were included as a result of the agreement with inclusion criteria described below.

Articles were excluded if they referred to the family or professional carers’ perceptions, included patients with exacerbations, included acute use of NIV or hospitalizations, did not report the COPD stage or did not analyse the direct effects of an intervention (e.g., study protocols). This permitted us to choose the articles most relevant to the aim of the study.

Articles further suggested by PubMed as relevant, citing the screened findings or found in references, were also checked. Each potential article was screened for sample characteristics, and included if it reported that: in the case of original research, the GOLD COPD criteria of severity stage III (or C) or higher, concerned at least 50% of participants; or in the case of a review, the GOLD COPD criteria of severity stage III (or C) or higher concerned 50% or more of the included studies.

**Figure 1:**
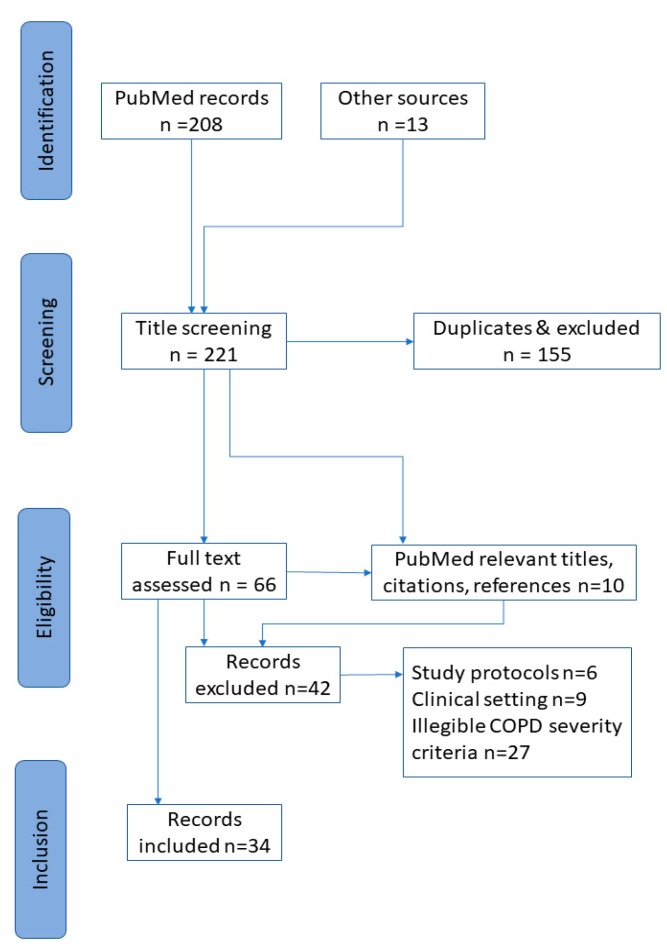
Flow chart of the literature search.

Two large studies were included despite the lower rate of severe COPD, for the effect of illness severity was included in the statistical analysis [13,14]. Ultimately, 34 articles were identified, representing qualitative, quantitative or mixed methods or review studies. They adopted a wide range of methodologies and outcomes, discouraging systematic comparisons. The process of articles’ selection is illustrated in the flow chart below (Figure 1).

## 3. Results

Psychosocial interventions aimed at improving the functioning—physically, psychologically or both—of patients with severe COPD are framed within various aims and means. We recognize that many issues might overlap yet be incomparable. For the clarity of the review, psychosocial interventions, in a comprehensive understanding of the term, were divided into four conventional thematic categories:

1. Home medical support;
2. Promoting self-management;
3. Framed to tackle low physical activity;
4. Focused on psychological comorbidities and impaired well-being/quality of life.

The categories were developed by authors assent after assessment of the content of all the articles, and were not prespecified in advance. The main characteristics of included research in terms of study design, intervention type, outcome, overlap and main limitations are presented in Tables 1–4 respectively.

To the best of our knowledge, there are only isolated studies that describe psychosocial interventions designed for patients treated with LTOT/NIV [15,16] and according to their needs [17]. Nevertheless, some studies included patients that receive such treatment [18], while others reported only on participants with severe/very severe COPD.

### 3.1. Home Medical Support

We identified 10 research efforts with the specific general medical care intervention, which addressed the psychosocial and clinical outcomes. Studies are briefly presented in Table 1. In seven studies the intervention consisted of comprehensive holistic care, namely palliative, which focused on HRQoL, rather than the improvement of survival. In three other studies, the care was enhanced with the telehealth tools: monitoring and assistance. The subjective appreciation of the intervention by patients was positive in most studies; however, objective measurement of the outcomes was not enough satisfactory. The most important outcome, HRQoL, was not improved in any of the studies. Ambiguous results were found in terms of hospitalization and exacerbation rate. Segrelles et al. showed reductions in emergency room visits, hospitalizations and their length, and the need for NIV. Moreover, the time to first severe exacerbation was doubled [16], but in the study of Janssens et al., a trend of higher hospital admissions in the intervention group was found [15].

#### 3.1.1. Palliative Care

With complex interdisciplinary treatment, palliative care is a good example of a comprehensive understanding of a psychosocial intervention. However, compared to palliative care for malignant illnesses, these services are still not perceived as an attainable standard for patients with severe COPD [25]. The lack of palliative care for COPD patients goes against the fact that the restraints of abilities, the needs and the health status in end-stage COPD often meets the same qualification criteria [12,17,26].

Recent studies brought a basic taxonomy of palliative care in advanced COPD [27]. Still, some unresolved issues remain and need to be addressed if palliative care is to be standardised and widely recommended [7,15,19]. The early introduction of palliative care is under investigation, as studies so far identified existing barriers, but not necessarily particular benefits for patients [18]. In a recent study with 69% of participants on LTOT, all having GOLD COPD stage III or IV, the introduction of palliative care for 12 months had no significant effects HRQoL, mood, symptoms report, admissions to hospital or presentations to emergency wards [15].

Similarly, introducing proactive palliative care—early introduced support from a specialised palliative care team, but not experienced, e.g., with respiratory failure—had no effect on the patients’ general HRQoL. Participants in the intervention group admitted, however, a lessened impact of COPD symptoms and a more positive attitude towards advanced care planning at some point during the intervention [19]. Importantly, recollection of participating in an early palliative care intervention for severe and very severe COPD patients was poor [7]. In turn, an intervention that was highly valued by patients suffering from breathlessness was based on integrative respiratory and palliative care that assured active symptom management, individualised education and advanced care planning [21]. More than half of the participants believed that their confidence and management of breathlessness had improved, they had been listened to carefully, and appreciated the continuity and long-term nature of care. A similar study with a randomised controlled trial (RCT) design [22], revealed that early palliative care, enhanced with breathlessness support service, might improve mastery and six-month-survival for patients with COPD. Both studies point out the role of specialist respiratory knowledge and experience joined with the standards of interdisciplinary palliative care.

#### 3.1.2. Telehealth

There is some evidence that patients might appreciate telehealth care [16]. Interventions for patients with GOLD COPD stage III or higher showed reduced numbers of acute exacerbations, hospitalizations and needs for NIV. Similarly, a review (not included) showed that telehealth might lead to a reduction in hospital admissions and emergency room visits, but not improve the HRQoL or mortality rates [28]. However, illness stage was not considered here. Another study found that telehealth monitoring was not a cost-effective tool, as it lacked many of the expected beneficial effects on depression, general HRQoL or the use of medical care, with an increase in the level of anxiety observed [29]. Since the results are somehow contradictory, a careful planning and evaluation of new technologies use in this group of interventions is required.

- Current results do not give a clear picture of whether home medical support added to standard therapy can have an influence on psychosocial functioning;
- Palliative care enriched with specialist respiratory elements can better attain the goals, in terms of coping with breathlessness and satisfaction with care, than for palliative care alone;
- Measures of HRQoL remain unchanged post-intervention, despite home medical support in various forms was analysed;
- Integrated palliative-respiratory care or telemonitoring can positively influence patients’ health status and reduce health care use, although ambiguous results were found in terms of hospitalization and exacerbation rate.

### 3.2. Self-Management

Self-management interventions aim at motivating, engaging and supporting patients for a positive adaptation of health behaviours, and a development of competences, easing disease management [30]. Supporting a patient’s self-management is often the priority amongst psychosocial interventions; sometimes it is constituted of an element of a complex pulmonary rehabilitation or medical care. Setting universal frameworks for effective encouraging COPD patients towards self-care is difficult. Severe COPD brings even more obstacles to overcome and less efficacy to be expected, often discouraging intervention development and implementation [31,32]. Table 2 provides an overview of studies on self-management intervention that reported on at least a majority of patients (or the majority of studies, in the case of a review) with advanced COPD.

A supported self-management intervention based on self-regulation theory and the “Living well with COPD” program was provided by a trained nurses and compared with a control group [33]. No intervention effect was found in terms of hospital readmission or death due to COPD or other causes, neither by the COPD stage or by intervention effect. A positive effect of the intervention on the illness impact dimension of HRQoL scale, with a trend of the effect on the total score, was found. Anxiety and depression scores improved at 12 months, yet without any intervention effect. Being classified as a successful self-manager post-intervention was related to a lower risk of readmission, and a longer time to exacerbation. In turn, minimal psycho-educative intervention dedicated for patients with advanced COPD was shown to improve the patients’ feelings of mastery, leading the authors to the conclusion that even in severe COPD, an enhancement of self-management abilities is a feasible task [34].

On one hand, a comprehensive analysis of self-management interventions for patients with moderate to severe COPD showed that improvement might be achieved in terms of behaviour change (12 out of 21 studied effects), self-efficacy (three out of seven effects) and HRQoL in some cases [35]. On the other hand, a confusingly high variety of self-management definitions, interventions’ compositions, sample selections and outcome measures, were demonstrated. Hence, the included studies were seen as underpowered to establish the meaning of particular interventions’ components or compositions. There were no observed effects on mood disorders [35].

In terms of HRQoL, self-management interventions were found to be especially effective for patients with severe symptoms, with no effect among those with moderate symptoms [36]. The same study reported an additional beneficial effect on emergency department visits, which was independent of the illness severity. Another review [13] examined the effects of self-management interventions enriched with action plans for COPD exacerbation. The results showed limited medical benefits—less admissions attributed to respiratory causes, but no effects on emergency department visits, hospitalisations, exacerbations or mortality. However, positive effects on HRQoL were acknowledged.

#### Teleinterventions

Self-management intervention for moderate to very severe COPD, enriched with digital monitoring, improved HRQoL, and showed a trend for a lower number of medical visits, depressive symptoms and improved specific health status [37]. This intervention was an Internet linked platform implemented on a computer. It provided monitoring and self-management support in terms of detecting exacerbations, compliance with inhaling medication, and enhanced psychological well-being.

Another intervention for patients with advanced COPD was based on telephone delivered health-coaching, inspired by motivational interviewing. It revealed positive effects on some dimensions of HRQoL and/or dyspnoea for the majority of participants [38]. An analysis of the role the illness severity played in the above effects would complement this report. In a review (not included) on e-interventions aiming at self-management in COPD, the authors [39] reported no adverse events and potential benefits for HRQoL and physical activity. However, they found no description of the severity of COPD among the investigated studies, which is what prevented planned subgroup comparisons. Since self-management and HRQoL improvement might be of particular importance for “home-tied” patients, the use of new technologies should be intensively researched on more homogenous samples of patients under LTOT/NIV.

- HRQoL and mastery in coping with dyspnoea can be improved by the majority of self-management interventions, especially among patients with the most advanced stage of illness;
- In some self-management programs, a reduction in hospital/emergency admissions and mental health problems were related to the intervention;
- Programs having a single provider, including elements of mental health care and offering multiple sessions, appear more effective;
- Internet or telephone-based interventions are promising means of enhancing self-management—particularly for patients who are not able to leave their homes.

### 3.3. Physical Activity

Studies examining the effects of physical activity among patients with advanced COPD are briefly presented in Table 3. Rehabilitation and exercise interventions, although well represented among patients with advanced COPD, are still rarely examined for those using LTOT/NIV. The participants in this population are seen as more difficult to recruit and less likely to complete the program [40]. A valuable verification of this opinion is a study done by Greulich et al. [41] with a large sample of patients suffering from very severe COPD (*n =* 554), showing that three weeks’ rehabilitation might improve patients’ physical functioning, including dyspnoea and well-being. An important result is that this intervention was especially effective for those that were treated with LTOT and had worse baseline characteristics.

Since patients with severe COPD often need additional air supply, Ricci et al. [42] reviewed benefits from adding NIV to a standard physical activity program. The results demonstrated the limitations of the studies included, and their ambiguous findings. A recent review of the same composition of methods [43] showed the benefits in terms of exercise capacity increase, dyspnoea decrease and better oxygenation, still warranting further research to develop the most effective standards. Another suggestion is that pulmonary rehabilitation (PR) being applied even once a week, but not once a month, can bring about significant changes in walking distance and some improvement in HRQoL [44].

Of note, a large study investigating PR [40] showed, apart from lowered dyspnoea and better exercise capacity, decreased anxiety and depression, plus an increase in HRQoL. The effect was observed despite the fact that patients with more severe airflow limitations and higher dyspnoea were less likely to complete the program. In turn, a relapse was observed in improved post-intervention HRQoL and depression scores six months post PR—compared to four weeks post PR, but not to baseline—with a particularly high risk of this effect for patients that did not live alone [51].

Contrary to the above findings, no effects of manual therapy on lung function or HRQoL was stated, and the therapy results for exercise capacity were found to have potential, but are too inconclusive so far [45]. HRQoL stays unchanged also, after upper limb exercise training, though upper limb endurance capacity increase and dyspnoea reduction was noted [48]. Interestingly, in another study, the size effect of physical improvement post PR was mediated by mental strategies of self-distracting, planning and active coping [52].

#### New Technologies Directed on Physical Activity

The use of new technologies in promoting physical activity has received increased scientific attention that has also reached patients with advanced COPD. Telerehabilitation was tested in Canada with the aim to increase PR accessibility and showed positive effects on exercise capacity and HRQoL [46]. Group PR delivered online might play an important role in also tackling patients’ social isolation and reach those in which transportation is a barrier in participation [49].

- Interventions based on physical activities are feasible even for the patients with very severe COPD, and boost positive changes;
- Programs incorporating physical exercises can be considered as remedies helping to alleviate psychological comorbidities;
- The issues of ensuring effective recruitment and preventing drop-out should be carefully addressed in all PR interventions.

### 3.4. Mental Health, Well-Being and Quality of Life

Not surprisingly, the research on interventions targeting anxiety and/or depression in patients with COPD are well represented, for these disorders are common comorbidities in COPD [53]. However, the knowledge about effective psychosocial support for patients with severe COPD and respiratory failure is particularly limited in this area. Interestingly, a scrupulous study on the treatment of anxiety and depression in COPD showed far more articles on the benefits of PR for advanced COPD than those which investigated the effects of cognitive-behavioural therapy [54]. The studies included are briefly presented in Table 4.

Psychological interventions, with a bias toward cognitive-behavioural therapy, are most often reported in relation to the mental health and well-being of COPD patients. Other interventions are life style oriented or incorporated into PR. Some comprehensive reviews analysing the interventions’ effects on anxiety and depression have been provided, yet studies including patients with advanced COPD are again a minority, and/or illness progress is usually not analysed as a potentially mediating factor [54,55,56,57], making this work inconclusive for the present subject. Though, it should be acknowledged that in some studies when severity of COPD was included in the analysis, the effects of the interventions on psychological and/or physical outcomes were independent of illness stage [14,50]. In one of those reviews, psychological interventions were found to have significant positive effects on depression, anxiety, HRQoL and dyspnoea, but not on the physical parameters of health status. The limitation is that no information was given whether the proportion of COPD grades was representative [14].

**Table 4:** Interventions in the category of mental health and well-being—first author, study design, characteristics of intervention, study overlapping area, main outcomes, dyspnoea, severity of COPD and limitations.

Study	Design	Specific Intervention	Overlapping with	Specific Outcomes or Conclusions for Reviews	Dyspnoea	COPD Stage, LTOT/NIV	Limitations
Farver-Vestergaard et al., 2015 [14]	Systematic review and meta-analysis	Psychological interventions for psychological and physical health outcomes	Medical care Exercise	The effect on anxiety, depression, dyspnoea and HRQoL was significant, a trend for exercise capacity and no effect on lung function and fatigue. Severity of COPD showed no effect on outcomes.	---	7 of 20 studies FEV1% M < 50% or 50% participants with severe COPD.	
Tselebis, 2016 [54]	Systematic review (part on PR programs only) *n* = 31	Interventions for anxiety and depression in COPD	Exercise	Patients with mild to severe mood symptoms might benefit more from PR. For advanced COPD, psychological support and dyspnoea management should be incorporated along with additional means to improve PR completion.	---	18 of 31 studies on comprehensive PR	
Bove et al., 2017 [58]	Qualitative study, *n* = 29	Home-based minimal psycho-educative intervention— patients experiences		Self-management support was perceived as supportive by enhancing internal resources which further helped to control the experience of anxiety and dyspnoea. The intervention also induced relief trough possibility to discuss end of life issues, and a feeling of being cared for.	mMRC M = 4.1 (3–5)	All C or D classification with GOLD; FEV1% M = 32% (14–57)	
Norweg and Collins 2013 [59]	Review of RTCs with dyspnoea as outcome *n* = 23	The mind-body interventions designed to alleviate dyspnoea	Medical Exercise Self-Management	Insufficient support for mind-body interventions to recommend, but promising effects in terms of anxiety, dyspnoea, distress and impact. Cognitive-behavioural therapy can influence affective dimension of dyspnoea through brain mechanisms. Slow-breathing can be recommended with possible similar effect of singing. Self-management programs have positive effect on dyspnoea even if served alone, and application of new technologies should be researched in this area.	Various measures	14 out of 23 studies included patients with FEV1% M < 50%	Variety of interventions and lack of dyspnoea index

Abbreviations: FEV1—forced expiratory volume in the first second, HRQoL—health related quality of life, LTOT—long-term oxygen therapy, M- mean, mMRC—modified Medical Research Council dyspnoea scale, *n*—number of subjects, NIV—non-invasive ventilation, PR—pulmonary rehabilitation, RCT—randomized controlled trial.

There are, however, examples of promising findings from tailored research. Among patients suffering from both severe COPD and anxiety, a reduction of anxiety symptoms was obtained in randomised controlled trial research with the use of a minimal psychoeducative intervention [34,58]. The observed effect was above the minimal clinically important difference. The intervention, rooted in cognitive-behavioural therapy, disclosed the relationships between thoughts, emotions, sensory perceptions and behaviours, permitting the thoughts related to anxiety and dyspnoea to be reframed.

A review of widely understood cognitive behavioural intervention strategies [59] showed similar positive effects (of various strength) on sensory and affective perception of dyspnoea in COPD. The change is regarded in terms of modification of perception, reduction of emotional distress, and resulting physiological and behavioural responses. Interventions included were: psychotherapy, distractive auditory stimuli, yoga, dyspnoea self-management education and slow-breathing exercises.

Another type of intervention, namely, motivational health coaching, was observed to improve self-rated health and HRQoL domains of emotional function, mastery and fatigue post-intervention, along with decreased dyspnoea 3 and 6 months later [38]. Motivational interviewing served as a base intervention concept. Each encounter emphasised the participant as the expert and the health coach as a good listener, helping the patients to adopt behaviours they perceived as a condition for pursuit of the treatment. Elements of self-management, and some exercise modelling, was also a part of the intervention. At each end point, at least 25% of patients achieved a clinically significant improvement 3 months post intervention.

A positive effect on well-being, emotional problems or HRQoL is also described in terms of PR [40,41,50,51]. The lack of homogeneity in samples and methods, however, prevents conclusions about particular exercises, programme complexity, length or specific groups of patients, and warrants further research. Interestingly, patients using active coping strategies (mental or behavioural technics that one uses when facing a stressful situation) were suggested to have improved effects of pulmonary rehabilitation [52]. The direction of this relationship needs a stronger confirmation.

In another area, ambient music added to a standard PR session for patients with advanced COPD was observed to have positive effect, with a reduction of anxiety that was noticed after one session [60]. Despite a small sample size, this finding appears promising.

Of importance, interventions regarding the spiritual well-being of patients with severe COPD were not found. In advanced COPD, patients’ well-being is similar to those with inoperable lung cancer, and dedicated support is essential in palliative care. Moreover, in severe COPD, the symptoms of dyspnoea are the strongest predictors of spiritual well-being, after allowing for factors such as age, marital status and HRQoL [9]. This dimension of patients’ functioning still waits for further research and the development of effective interventions.

Surprisingly, none of the included studies intentionally analysed any form of intervention’s impact on the quality or quantity of patients’ interpersonal relationships. We consider it a gap which necessarily should be complemented to diminish the feeling of isolation. It often accompanies those patients who spent tens of hours a week tied down to their home oxygen treatment or ventilatory support. Moreover, improvement in social functioning can further prevent or alleviate mental health problems.

All studies included in this section showed beneficial outcomes. Only one review recognised the potential role of COPD stage, but found no effect; none reported the proportion or the variance analysis in terms of respiratory failure.

- This group of interventions have positive effects on emotional problems and at least some dimensions of HRQoL;
- The most promising results are achieved when mental health care is integrated with an exercise program;
- Cognitive-behavioural therapy, mindfulness, psycho-educative and mind-body interventions show at least partial evidence for efficacy to target mental health problems;
- Studies including patients with severe COPD are underpowered to provide detailed conclusions.

## 4. Discussion

### 4.1. Review Findings

The results of the literature search permitted a comprehensive overview on accessible psychosocial actions aimed at the improvement of functioning among patients suffering from severe COPD. We found that home medical support gains in efficacy when enriched with specialist respiratory care or telemonitoring. Self-management interventions appear to have a wide impact, improving HRQoL, emotional functioning and health status. Both self-management and exercise programs are able to reach most affected patients with severe COPD (also those with respiratory failure), improving their psychosomatic condition. Tackling mental health and well-being in this group of patients seems underrepresented in current literature. Since there is a large rate of research with overlapping categories, the development of multimethod approach tailored to the abilities and needs of those suffering from severe COPD is warranted.

### 4.2. Limitations

The present study has some limitations. A wide range of intervention forms was collated, which prevented a detailed analysis of particular methods or interactions between them. The proposed division of the articles into four separate themes was arbitrary and might have resulted in the omission of some phenomena. For example, a use of new technologies for this group of patients could possibly be seen as an independent issue. Using a cut-off point of 50% of patients with severe COPD participating in included articles possibly caused a bias in the findings analysed toward patients with better health conditions. This review points out more on the directions for future research than it finds answers on practical questions concerning psychosocial care for this group of patients.

### 4.3. Further Research

Future studies should consider few directions. The precise analysis of patients’ illness stages and the treatment role in all interventions’ assessments are urgent needs that warrant quality, evidence-based data, which is unavailable currently. COPD patients with distinct therapies have experiences characteristic of their treatment group [6,7,61]. Thus, it is difficult to understand the methodological approach in some studies where, despite a small sample size, the patients’ treatments, or even COPD stages, are neither provided nor included in analyses [57,62]. This, in our opinion, is the main factor limiting the identification of publications where, e.g., patients with respiratory failure, are included.

Gaps in patients’ knowledge are widely stated and perceived as barriers in using palliative care [7], thus it is somewhat surprising that only a couple of recent interventions pointed out patients’ education [34,58] compared with studies two decades ago [63]. It appears that psychoeducative intervention presents no conflict with programs of physical activity in terms of aims or feasibility, or with interventions helping patients by providing early palliative care joined with respiratory care. Many of the pulmonary rehabilitation programs show effectiveness resulting from the integration of exercise, psychosocial support and education [42], some of those being dedicated to patients with respiratory failure [41,43]. Nevertheless, further research investigating the effects of both multidisciplinary, specific interventions and including representative groups of participants with advanced stages of COPD is warranted.

Despite the fact that methodological differences should be recognised first, the above review of interventions introducing palliative care for patients with severe COPD points to the role of education and improvement through self-management. Enhancing patients’ self-efficacy might be a key to boosting the applicability and patients’ adaptation [64]. Among patients with COPD stage III to IV, a higher level of coping techniques referring to as planning strategies, self-distraction and active coping, predicted benefits from PR [52]. According to Boutou [40], patients who completed PR differ from non-completers not by body-mass index or exercise capacity, but by FEV1% predicted and better baseline scores in mastery, depression, anxiety, and total HRQoL. To some extent, coping strategies or self-efficacy can be trained, thus, including both into psychosocial interventions is worth considering. Two issues complementing the above list of desired research among patients affected by severe COPD are interventions addressing social isolation and those addressing spiritual well-being.

## 5. Conclusions

The knowledge about psychosocial interventions dedicated to patients with severe COPD, especially with chronic respiratory failure, is scarce. In recent studies, the focus on self-efficacy, telehealth and physical activity in both physical and mental health preservation is noticeable. Combined palliative and respiratory care could be considered most beneficial in terms of end-stage COPD. There are many paths for future research—from detailed explorations of the roles the stage and treatment of the illness are playing, through to new educational approaches: the use of modern technologies to enhance social and spiritual dimensions of patients’ functioning.

## Figures and Tables

**Table 1:** Interventions in the medical care category—first author, study design, characteristics of intervention, study overlapping area, main outcomes, dyspnoea, severity of COPD and limitations.

Study	Design	Specific Intervention	Overlapping with	Specific Outcomes or Conclusions for Reviews	Dyspnoea	COPD Stage, LTOT/NIV	Limitations
Véron et al., 2018 [7]	Retrospective qualitative study *n* = 19	Early palliative care group	---	Identification of barriers hindering application of palliative care in severe COPD: Poor recollection, lack of understanding of the purpose of intervention, denial of being ill, restrictions in daily activities (often due to oxygen therapy), anxiety and helplessness	---	FEV1% M = 35.4% (SD = 18.6) plus LTOT/NIV	retrospective
Janssens et al., 2019 [15]	Prospective controlled randomised (pilot) study, *n* = 49	Early palliative care group	---	A trend of higher hospital admissions in intervention group; no effect on HRQoL measurement, or on mood disturbances. Subjectively 6 months post, 94% patients in intervention appreciated the care, 50% perceived positive impact on HRQoL	---	III/IV stage plus LTOT or NIV	Small n
Segrelles et al., 2014 [16]	Open-label, controlled, non-blind clinical trial n = 60	Telehealth— everyday self-monitoring and medical assessment of basic health		Reduction in emergency room visits, hospitalizations and its length, and need for NIV. The time to first severe exacerbation doubled. High level of patients’ satisfaction.	mMRC M = 1.8 (SD = 1.2)	FEV1% M< 50%, all on LTOT	
Horton et al., 2013 [18]	Single-centre cohort prospective study, *n* = 30 patients, 18 caregivers	Palliative care trial	Self-management Well-being	Symptoms severity, caregiver burden and HRQoL stayed unchanged. Despite the preference to die at home, 16 patients died in hospital during the study period. Palliative care was valued by participants.	mMRC = 5 for 60% patients	Severe COPD or moderate with poor prognosis; 15 patients on LTOT	No control group
Duenk et al., 2017 [19]	Quasi experimental (pragmatic cluster controlled trial) *n* = 228	Proactive palliative care	Well-being	No effect on general HRQoL or mood at 3, 6, 9, 12 months. Intervention group reported less impact of COPD at 6 months and had made advanced care planning choices more often. No effects on readmissions or survival.	mMRC 78.1% ≥ 4	65.8% with III/IV stage COPD—all with poor prognosis	Possible bias
Schroedl et al., 2014 [20]	Retrospective case series, *n* = 36	Outpatient palliative medicine program	Self-management Well-being	Palliative care can adequately address illness burden by increasing advanced planning, and accompanying physical and psychological symptoms which were not treated before intervention		72% had III-IV GOLD stage; 72% on LTOT	retrospective
Smallwood et al., 2019 [21]	Retrospective questionnaire; n=64 patients and 24 carers	Integrated respiratory and palliative care		High patient satisfaction (e.g., advice, time scheduling, felt being cared for). 76.6% felt definitely more confident self-managing symptoms	mMRC Md = 3 (2–4)	Fev1% Md = 40% 31-50); 56.3% home oxygen use	No comparison group
Higginson et al, 2014 [22]	Single-blind RCT, *n* = 105	Integrated palliative and respiratory care – breathlessness support service	Well-being	Improved mastery in breathlessness management at 6 weeks; higher survival rate at 6 months. No effect on breathlessness, HRQoL, depression or anxiety nor for days in hospital since readmission at 6 weeks.	On scale 0–10, last 24h M = 5.9 (SD = 2.0)	Mixed illnesses, 54% with COPD; FEV1% (for all) M = 46.2% (SD = 23.3).	Not a homogenous sample
Vitacca, Comini et al., 2019 [23]	Prospective observational study n = 10	Advanced care planning with tele-assisted support for palliative care	Well-being	Patients acceptance and maintaining stable level of anxiety, plus addressing patient’s problems with negative emotions, bad days and illness deterioration during 6 months was noted.	--	All patients with severe COPD	No comparison group
Lewis et al., 2010 [24]	Randomised trial followed by a passive period, n = 40	Telemonitoring vs standard care	Well-being	No changes in QoL or emotional functioning was observed during or at the end point of intervention.	mMRC M = 3.5	FEV1% M = 39%	

Abbreviations: FEV1—forced expiratory volume in the first second, HRQoL—health related quality of life, LTOT—long-term oxygen therapy, mMRC—modified Medical Research Council dyspnoea scale, M—mean, Md—median, *n*—number of subjects, NIV—non-invasive ventilation, PR—pulmonary rehabilitation, RCT—randomized controlled trial, SD—standard deviation.

**Table 2:** Interventions in the self-management category: first author, study design, characteristics of intervention, study overlapping area, main outcomes, dyspnoea, severity of COPD and limitations.

Study	Design	Specific Intervention	Overlapping with	Specific Outcomes or Conclusions for Reviews	Dyspnoea	COPD Stage, LTOT/NIV	Limitations
Lenferink et al., 2017 [13]	Systematic review of RCTs *n* = 22	Self-management interventions with action plans for exacerbation of COPD	HRQoL Medical care	Positive effects on HRQoL—not clinically relevant, and respiratory-related hospital admissions. No effect on emergency room visits, hospitalization, dyspnoea, exacerbations or mortality, with a trend of higher respiratory-related mortality in intervention groups.	Baseline n/a. mMRC as outcome in 3 studies—range of means from 1.1 to 3.6	In 10 out of 22 studies FEV1% M < 50%	
Bucknall et al., 2012 [33]	RCT, *n* = 464	Training in detection and promptly treating of exacerbations + 12mths ongoing support	Medical care HRQoL	No effect on readmissions to hospital or death due to COPD; no differential effect in relation to COPD stage or demographics; post-intervention successful self-managers (42% participants—younger and not living alone) had lower risk of readmission or death.	---	FEV1% M = 40.5 (SD = 13.6) 34 with LTOT	
Bove et al., 2016 [34]	RCT, *n* = 66	Psychoeducational intervention for patients with comorbid anxiety, inspired by cognitive-behavioural therapy	Well-being	Normalized reaction for dyspnoea. Reduced anxiety and increased mastery assessed by the Chronic Respiratory Questionnaire	mMRC M = 4 (range 3–5)	C/D GOLD, FEV1% M = 34.0% (SD = 13.2), 13 on home oxygen	Small sample
Jordan et al., 2015 [35]	Systematic review (1 and 4) —Cochrane	Single component and composed interventions for self-management improvement	Well-being Medical care	Structured exercise enhances multicomponent interventions. Interventions might have positive effect on hospital admissions (if including enhanced care and support), HRQoL and dyspnoea reduction.	Measurement heterogeneity	Moderate to severe FEV1% M = 35.8% (SD = 7)	
Newham et al., 2017 [36]	Systematic review with meta-analysis, *n* = 24	Review of Self-management interventions reviews in relation to the effect on HRQoL.	Well-being Medical care	HRQoL improved only in samples of patients with severe but not moderate symptoms. Interventions were effective with single provider, multiple sessions and when targeting mental health. Less ED admissions were noted for intervention groups, independent of illness stage.	---	14 of 24 studies had participants with advanced COPD	No data about the COPD severity and its indicators
Farmer et al., 2017 [37]	RCT *n* = 166	Digital self-management support	Well-being Medical care	No effect of adding digital monitoring and self-management on specific health status. A trend towards less medical visits, symptoms of depression and hospitalizations was observed. HRQoL improved.	mMRC 3–68.5%; 4–14.9%	Severe or very severe COPD–60.8%	
Rehman et al., 2017 [38]	Prospective intervention study, *n* = 50	Telephone based intervention of health-coaching rooted in motivational interviewing	Self-management	HRQoL improved in terms of fatigue, emotional function, mastery and health self-rating.	mMRC M = 2.4 (SD = 1)	FEV1% M = 39% (SD = 15)	Unclear study design in terms of RCT

Abbreviations: ED – emergency department, FEV1—forced expiratory volume in the first second, HRQoL—health related quality of life, LTOT—long-term oxygen therapy, M—mean, mMRC—modified Medical Research Council dyspnoea scale, *n*—number of subjects, NIV—non-invasive ventilation, RCT—randomized controlled trial, SD—standard deviation.

**Table 3:** Interventions in the category of physical activity—first author, study design, characteristics of intervention, study overlapping area, main outcomes, dyspnoea, severity of COPD and limitations.

Study	Design	Specific Intervention	Overlapping with	Specific Outcomes or Conclusions for Reviews	Dyspnoea	COPD Stage, LTOT/NIV	Limitations
Boutou et al., 2014 [40]	Prospective, multicentre, *n* = 787	Pulmonary rehabilitation program	Well-being, medical	Patients most likely to benefit from PR appeared the least likely to complete it. Alleviation of dyspnoea and higher exercise capacity. Lower depression and anxiety scores, improved HRQoL	mMRC M = 3.3 (SD = 0.9) for all	51.2% COPD stage ≥ III FEV1 % M = 49.7 (SD = 19.7) for all	
Greulich et al., 2015 [41]	Retrospective analysis, *n* = 554	In-house pulmonary rehabilitation program	Well-being	Reduction of dyspnoea, improved well-being and physical functions. Patients on LTOT benefited more on HRQoL than those not on LTOT. Worse baseline scores were related to most benefits post-intervention.	mMRC M = 3.17 (SD = 1.14)	Stage IV COPD; FEV1% M = 34.2, (SD = 7.7); 60% on LTOT	No usual care control group
Ricci et al., 2014 [42]	Review and meta-analysis, *n* = 8	Physical activity with oxygen/NIV provision	Medical care	Using NIV during physical exercises could not be confirmed as superior to usual training, still training extension or intensity might improve exercise capacity.	---	FEV1% mean from 26% to 48%	Underpowered for methodological reasons.
Vitacca and Ambrosino 2019 [43]	Narrative review	Addition of NIV to standard exercise training	---	Improvement in exercise tolerance + for patients on home ventilatory support NIV during exercise can improve oxygenation and diminish dyspnoea.	---	Patients with respiratory failure	
Chigira et al., 2014 [44]	Comparative prospective observational study, *n* = 36	Comparison of PR taken once a week vs once a month	HRQoL	No change in respiratory function. Higher frequency of PR resulted in longer 6MWT and slightly increased average HRQoL	---	GOLD stage III *n* = 13, IV *n* = 11	All patients independent in activities of daily living
Simonelli et al., 2019 [45]	Review of RCTs, *n* = 6	Effectiveness of manual therapies	Medical care	Potential effect on exercise capacity was found with other findings being too weak to prove or exclude the effect on HRQoL or lung function.	---	4 of 6 studies FEV1% M < 50%	All studies assessed as having high risk of bias
Marquis et al., 2015 [46]	Pre-experimental study, *n* = 26	In-home telehealth pulmonary rehabilitation	Self-management Well-being	Improvement was found in: 6MWT, cycle endurance test and HRQoL domains of dyspnoea, fatigue and emotion but not mastery.	---	FEV1% predicted M = 47.7%, GOLD severe +61.5%	small n
Braz Junior et al., 2015 [47]	Pilot study, *n* = 11	Whole-body vibration for severe COPD	HRQoL	The use of vibration platform training increased 6MWT distance and HRQoL	---	FEV1% M = 14.6% (SD = 11.1)	small n
McKeough et al., 2016 [48]	Systematic review (Cochrane), *n* = 15	Upper limb exercise training for COPD	---	Dyspnoea and endurance upper limb capacity but not HRQoL can be improved with some form of upper limb exercise in comparison with no training or sham intervention.	---	11 of 15 studies with patients with advanced COPD.	Low to moderate quality of studies mostly due to small n.
Burkow et al. 2015 [49]	Mixed methods pilot study, *n* = 10	Online home-based comprehensive group pulmonary rehabilitation	Self-management	This intervention is feasible and acceptable for patients being a source of knowledge and social support. HRQoL slightly improved, mainly on the *Impact of illness* dimension	---	4 of 10 grade III and 4 grade IV GOLD	Small n and inconclusive patients outcomes
Tselebis et al., 2013 [50]	Prospective observational study, *n* = 101	PR program (exercise only) with assessment of anxiety and depression	Well-being	Depression and anxiety were significantly reduced for patients at all COPD stages.	---	74 of 101 patients with severe or very severe COPD	Unclear study design
Bratås et al., 2012 [51]	Prospective observational study *n* = 111	PR program with assessment of anxiety, depression and HRQoL	Well-being	After the improvement of anxiety, depression and HRQoL 4 weeks post PR a significant decrease of all main outcomes was observed. Illness severity was not an intervening variable.	---	25.5% with III and 34.2% with IV stage GOLD	
Russo et al., 2017 [52]	Observational study *n* = 76	Effects of PR as mediated by coping strategies	Self-management	The degree of physical response to PR (6MWT) was related to self-distractive, planning and active coping strategies.	mMRC M = 4.0	Age 70+, GOLD III and IV stage	Only basic statistical analysis were provided

Abbreviations: 6MWT—six minute walking test—distance is measured in meters, FEV1—forced expiratory volume in the first second, HRQoL—health related quality of life, LTOT—long-term oxygen therapy, M—mean, mMRC—modified Medical Research Council dyspnoea scale, *n*—number of subjects, NIV—non-invasive ventilation, PR—pulmonary rehabilitation, RCT—randomized controlled trial, SD—standard deviation.
